# Lower Plasma IL-32 Levels Linked to Better Survival in Sepsis

**DOI:** 10.3390/biomedicines13030750

**Published:** 2025-03-19

**Authors:** Patricia Mester, Alexander Utrata, Niklas Schmidtner, Charlotte Birner, Stephan Schmid, Martina Müller, Vlad Pavel, Christa Buechler

**Affiliations:** Department of Internal Medicine I, Gastroenterology, Hepatology, Endocrinology, Rheumatology, and Infectious Diseases, University Hospital Regensburg, 93053 Regensburg, Germany; patricia.mester@klinik.uni-regensburg.de (P.M.); alexander.utrata@stud.uni-regensburg.de (A.U.); niklas.schmidtner@stud.uni-regensburg.de (N.S.); charlotte.birner@stud.uni-regensburg.de (C.B.); stephan.schmid@klinik.uni-regensburg.de (S.S.); martina.mueller-schilling@klinik.uni-regensburg.de (M.M.); vlad.pavel@klinik.uni-regensburg.de (V.P.)

**Keywords:** COVID-19, liver cirrhosis, sepsis, mortality

## Abstract

**Background/Objectives:** Interleukin-32 (IL-32) is a pro-inflammatory cytokine primarily produced by immune cells and involved in bacterial and viral infections. This study investigates whether plasma IL-32 is associated with sepsis severity and clinical outcomes. **Methods:** Plasma IL-32 levels were measured in 186 patients with systemic inflammatory response syndrome (SIRS), sepsis, or septic shock, as well as in 40 controls. The relationship between IL-32 levels and SARS-CoV-2 or bacterial infections, alongside underlying etiological conditions, was assessed. **Results:** Patients with liver cirrhosis exhibited elevated plasma IL-32 levels. After excluding these patients, IL-32 levels were lower in SIRS/sepsis patients compared to the controls. No significant differences in IL-32 levels were observed among SIRS, sepsis, and septic shock patients. Additionally, underlying conditions such as pancreatitis and cholangitis did not influence IL-32 levels. Patients with bloodstream bacterial infections, SARS-CoV-2 infections, or no documented infection had comparable IL-32 levels. Notably, higher IL-32 levels were associated with increased mortality. **Conclusions:** These findings suggest that a reduction in plasma IL-32 levels may be protective in SIRS/sepsis patients, as elevated levels are linked to poor survival outcomes.

## 1. Introduction

Sepsis is a life-threatening condition characterized by a dysregulated immune response to infection, leading to widespread inflammation, tissue damage, and organ failure [1,2]. A key feature of sepsis is immune dysfunction, including T-cell impairment and lymphopenia, which contribute to increased susceptibility to secondary infections and poor outcomes. Natural killer (NK) cells, a critical component of the innate immune system, play a complex role in sepsis. While some studies suggest a protective effect, others indicate that NK cell activation may contribute to inflammation and organ injury [2,3,4].

Interleukin-32 (IL-32), originally termed natural killer cell transcript 4 (NK4), is predominantly expressed in activated T cells and NK cells [5]. In the early stages of sepsis, NK cells become activated and release various cytokines, including IL-32, which can exacerbate inflammation and contribute to tissue damage [6,7]. However, the precise role of NK cells in sepsis remains uncertain, as both beneficial and harmful effects have been reported [7]. Giamarellos-Bourboulis and colleagues demonstrated that sepsis patients with elevated NK cell levels had improved survival compared to those with lower levels [8]. Conversely, Boomer et al. found that NK cell numbers in the peripheral blood of sepsis patients were significantly reduced, potentially increasing the risk of nosocomial infections and worsening clinical outcomes [9].

IL-32 is more abundant in immune cells than in non-immune cells and plays a pivotal role in inflammation. It has been shown to induce the expression of proinflammatory cytokines, such as tumor necrosis factor (TNF), IL-6, and IL-1β. IL-32 has been implicated in the pathogenesis and progression of several inflammatory diseases, including inflammatory bowel disease, Sjögren’s syndrome, rheumatoid arthritis, and chronic obstructive pulmonary disease [5,10,11]. In patients with chronic liver disease, hepatic IL-32 expression is elevated and correlates with disease severity [12,13,14].

IL-32 expression is upregulated in lung granulomas and the airway epithelial cells of patients infected with Mycobacterium avium. This cytokine has been shown to reduce bacterial load in macrophages and epithelial cells, suggesting a potential role in host defense [15]. However, the function and expression of IL-32 in other bacterial infections remain poorly understood. Further research is needed to elucidate its precise role in infection and immune regulation.

Emerging evidence suggests that IL-32 plays a significant role in antiviral immunity. This cytokine induces type I and type III interferon responses, which are crucial for host defense against viral infections. Elevated serum levels of IL-32 have been observed in patients with chronic human immunodeficiency virus (HIV) infection and in individuals infected with influenza A virus [16,17]. Similarly, IL-32 levels have been investigated in patients with Coronavirus Disease 2019 (COVID-19), an acute respiratory illness caused by severe acute respiratory syndrome coronavirus 2 (SARS-CoV-2). These findings indicate that IL-32 may contribute to the immune response during viral infections, though its precise role remains to be fully elucidated [18,19,20,21,22].

SARS-CoV-2 infection can lead to a respiratory syndrome associated with pneumonia, presenting with a wide spectrum of clinical phenotypes, ranging from mild to severe. In severe cases, patients develop acute respiratory distress syndrome (ARDS), often necessitating mechanical ventilation. Severe COVID-19 is characterized by an excessive release of pro-inflammatory cytokines, contributing to lung injury and multi-organ damage [23,24,25,26]. Several studies have investigated the role of IL-32 in COVID-19. One study reported elevated IL-32 levels in COVID-19 patients compared to healthy controls; however, IL-32 concentrations were not correlated with disease severity or survival. Notably, COVID-19 patients with fever had significantly higher IL-32 levels than those without [21]. Another study found positive associations between IL-32 levels, lung function, and survival in hospitalized patients with mild, moderate, or severe disease [19]. Despite these findings, IL-32 levels were not linked to mortality [20]. Conversely, a separate study observed decreased serum IL-32 levels in patients with mild, moderate, and severe COVID-19 compared to healthy individuals. In these cases, IL-32 levels were not associated with disease severity. Instead, interleukin-6 (IL-6) demonstrated high diagnostic accuracy for severe COVID-19 [18]. These conflicting findings highlight the need for further research to clarify the precise role of IL-32 in SARS-CoV-2 infection and its potential as a biomarker for disease progression

IL-32 is a pro-inflammatory cytokine that promotes endothelial cell angiogenesis and modulates lipid metabolism, linking it to cardiovascular disease. However, despite the increased risk of atherosclerosis in COVID-19 patients—particularly in severe cases—no clear association has been observed between IL-32 levels and cardiovascular disease in these individuals [20]. This raises the question of whether systemic IL-32 plays a role in viral infections and disease severity in COVID-19. Moreover, studies measuring circulating IL-32 in patients with severe inflammatory diseases are limited. In patients with severe community-acquired pneumonia—a condition frequently associated with acute respiratory failure and septic shock—plasma IL-32 levels were significantly elevated compared to healthy controls [27]. This suggests that IL-32 is involved in severe systemic inflammation. Additionally, IL-32 concentrations correlated with disease severity, vasopressor therapy requirements, and the need for invasive mechanical ventilation. Importantly, IL-32 was a stronger predictor of mortality than traditional inflammatory markers such as white blood cell count and C-reactive protein. Increased IL-32 expression in peripheral blood mononuclear cells may contribute to these elevated systemic levels [27].

The aim of our study was to analyze plasma IL-32 levels in a large cohort of critically ill patients to identify associations with underlying diseases, bacterial and SARS-CoV-2 infections, and disease severity.

## 2. Materials and Methods

### 2.1. Study Cohort

Between August 2018 and January 2024, plasma samples were obtained from 186 patients admitted to the intensive care unit at the University Hospital of Regensburg. Patients were categorized according to the Sepsis-3 criteria [28] and the systemic inflammatory response syndrome (SIRS) criteria [29] for sepsis, septic shock, and SIRS. Our patients had different causes of SIRS (50), sepsis (45), or septic shock (91). Our cohort included 28 sepsis/septic shock patients who were hospitalized for SARS-CoV-2 infection. Plasma samples from COVID-19 patients were collected between October 2020 and January 2023.

Patients with hepatitis virus infection, human immunodeficiency virus infection, or multidrug-resistant infections were excluded. Laboratory values were provided by the Institute of Clinical Chemistry and Laboratory Medicine at the University Hospital of Regensburg, and microbiological tests by the Institute of Clinical Microbiology and Hygiene at our hospital.

Otherwise, all patients admitted to the intensive care unit who were willing to participate and were categorized as described above were consecutively included in this retrospective study. All patients who left the intensive care unit alive were defined as survivors, and patients who died during their intensive care unit stay were defined as non-survivors.

The study protocol was approved by the ethical committee of the University Hospital of Regensburg (18-1029-101) and was performed according to the updated guidelines of good clinical practice and the updated Declaration of Helsinki. Informed consent was obtained from all subjects (or relatives on behalf of the patient) involved in the study.

### 2.2. IL-32 ELISA

Blood samples were collected from patients within 12 to 24 h of admission to the intensive care unit. Ethylenediaminetetraacetic acid was used as an anticoagulant. Plasma was aliquoted and stored at −80 °C until use. There are no studies having analyzed the stability of IL-32 during storage to our knowledge. The IL-32 levels of 20 patients whose plasma was collected early during this study and of 20 patients whose plasma was collected late during this study were similar (*p* > 0.05). This might indicate that IL-32 is relatively stable during long-time storage.

Plasma IL-32 levels were measured using a human IL-32 ELISA kit (order number DY3040-05, R&D Systems, Wiesbaden, Germany), following the manufacturer’s instructions. The assay range is 78.1–5000 pg/mL. Measurements were performed using undiluted plasma. Standard samples and plasma samples were measured in duplicate using the iMark^TM^ microplate reader from BIO-RAD (Munich, Germany), and the mean values were used for calculations. A representative standard curve is shown in Figure S1.

### 2.3. Statistical Analysis

Data are presented as box plots displaying the minimum and maximum IL-32 levels, the median, and the first and third quartiles. Outliers are represented by single circles or asterisks. Tables provide the median, minimum, and maximum values for each dataset. Statistical analyses were performed using IBM SPSS Statistics 26.0 (IBM Corp., Armonk, NY, USA; released 2019). IL-32 levels in plasma of SIRS/sepsis patients and controls were not normally distributed (*p* < 0.001 Shapiro–Wilk test for both) and the following tests were applied: (1) Mann–Whitney U test (non-parametric) for comparisons between two groups; (2) Kruskal–Wallis test (non-parametric) for comparisons between multiple groups; (3) chi–squared test for categorical variables; (4) Spearman’s correlation to assess relationships between continuous variables; and (5) receiver operating characteristic curve for the discrimination of survivors and non-survivors. A level of *p* < 0.05 was considered significant.

## 3. Results

### 3.1. IL-32 in Plasma of Controls and SIRS/Sepsis Patients with and Without Liver Cirrhosis

Plasma IL-32 levels were measured in 186 patients with SIRS/sepsis and 40 control subjects. Compared to the control cohort, the patients were older and had a higher proportion of females (Table 1).

**Figure 1:**
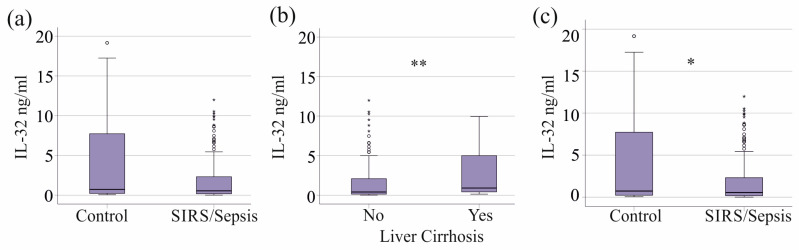
IL-32 in plasma of controls and SIRS/sepsis patients. (**a**) Plasma IL-32 levels of the 40 controls and the 186 SIRS/sepsis patients. (**b**) Plasma IL-32 levels of sepsis patients without (No) and with (Yes) liver cirrhosis. (**c**) Plasma IL-32 levels of the 40 controls and the 152 SIRS/sepsis patients after exclusion of patients with liver cirrhosis. Outliers are represented by circles and small asterisks. Mann–Whitney U test: * *p* < 0.05, ** *p* < 0.01.

Plasma IL-32 levels did not differ significantly between the control group and patients with SIRS/sepsis (*p* = 0.550, Figure 1a). However, patients with SIRS/sepsis and liver cirrhosis (n = 34) had significantly higher IL-32 levels compared to SIRS/sepsis patients without cirrhosis (*p* = 0.002, Figure 1b).

When patients with liver cirrhosis were excluded, the remaining 152 patients with SIRS/sepsis had significantly lower IL-32 levels than the healthy controls (*p* = 0.015, Figure 1c). Median IL-32 levels in patients with SIRS/sepsis and liver cirrhosis were 0.9 ng/mL (range: 0.1–10.0 ng/mL), comparable to those in the control group (0.7 ng/mL; range: 0–19.2 ng/mL; *p* > 0.05). In contrast, patients with SIRS/sepsis without liver cirrhosis had lower plasma IL-32 levels, with a median of 0.4 ng/mL (range: 0–12.0 ng/mL; Figure 1c).

These findings indicate that plasma IL-32 levels are reduced in patients with SIRS/sepsis, except in those with concurrent liver cirrhosis, where IL-32 concentrations are normal.

In patients with SIRS/sepsis, plasma IL-32 levels showed a significant negative correlation with age (correlation coefficient r = −0.219, *p* = 0.007). However, no significant correlation was observed between IL-32 levels and body mass index (BMI) (r = 0.098, *p* = 0.238). Plasma IL-32 concentrations were similar between male and female patients (*p* = 0.435).

### 3.2. Plasma IL-32 of SIRS/Sepsis Patients Stratified for SIRS, Sepsis, and Septic Shock and Underlying Diseases

**Figure 2:**
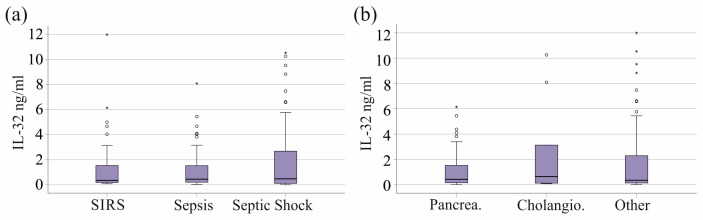
Plasma IL-32 levels of patients with SIRS, sepsis, and septic shock and association with pancreatitis and cholangiosepsis. (**a**) Plasma IL-32 levels of SIRS/sepsis patients categorized according to the SIRS criteria and to the Sepsis-3 definition. (**b**) Plasma IL-32 levels of SIRS/sepsis patients with pancreatitis (Pancrea), cholangiosepsis (Cholangio), and patients without these underlying diseases (Other). Outliers are represented by circles and small asterisks. Statistical test used: Kruskal–Wallis test.

Patients were divided into the categories of SIRS (39 patients), sepsis (37 patients), and septic shock (76 patients) [29]. Patients with septic shock were older than those with sepsis (Table S1). The number of immature granulocytes was the lowest in patients with SIRS (Table S1). Disease severity and the mortality of the patients increased from SIRS to sepsis to septic shock [30], and almost all of our non-surviving patients had septic shock (Table S1). There was no difference in circulating IL-32 levels between these groups (*p* = 0.968, Figure 2a).

In our cohort, 41 patients developed SIRS/sepsis due to pancreatitis and 9 due to cholangitis. The number of patients with these underlying conditions did not differ between SIRS, sepsis, and septic shock (Table S1). Patients with pancreatitis and cholangiosepsis had similar plasma IL-32 levels compared to all other patients (*p* = 0.765, Figure 2b).

### 3.3. Plasma IL-32 Levels of SIRS/Sepsis Patients Stratified for Infectious Diseases, SARS-CoV-2, and Bacterial Infections

**Figure 3:**
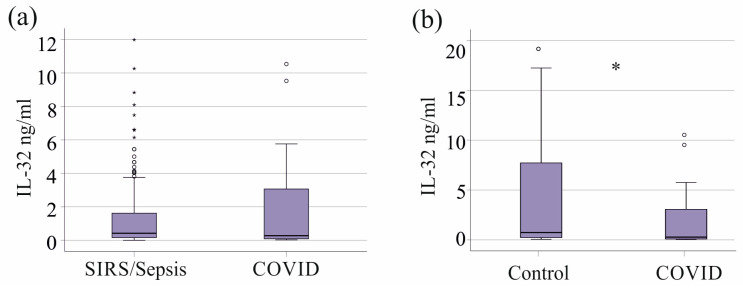
Plasma IL-32 levels of patients with SIRS/sepsis and COVID-19. (**a**) Plasma IL-32 levels of SIRS/sepsis patients without and with COVID-19. (**b**) Plasma IL-32 levels of the healthy controls and patients with COVID-19. Outliers are represented by circles and small asterisks. Statistical test used: Mann–Whitney U test, * *p* < 0.05.

SARS-CoV-2 infection has recently been recognized as a cause of sepsis [31,32], and all COVID-19 patients in our study presented with sepsis (7 patients) or septic shock (21 patients, Table S1). However, IL-32 levels were comparable between the 28 patients with severe COVID-19 and critically ill patients without this viral infection (Figure 3a). Interestingly, IL-32 levels were higher in the healthy controls than in patients with COVID-19 (Figure 3b).

The most common infections leading to SIRS/sepsis in our cohort were pulmonary infections (n = 43) and urinary tract infections (n = 16). Pulmonary infections were most common in patients with septic shock (Table S1). Plasma IL-32 levels did not significantly differ between these two groups (*p* = 0.843).

The plasma IL-32 levels of the 19 patients with Gram-negative bacteria, the 21 patients with Gram-positive bacteria, and the 4 patients with both types of bacteria in their blood cultures were similar to those with negative blood cultures (*p* = 0.621). The type of bacterial infection did not differ between SIRS, sepsis, and septic shock (Table S1).

### 3.4. Plasma IL-32 Levels in Relation to Vasopressor Therapy and Interventions

Plasma IL-32 levels were not associated with the need for dialysis, mechanical ventilation, or vasopressor treatment (Table 2), which were more commonly required in patients with septic shock (Table S1).

### 3.5. Plasma IL-32 Levels in Relation to Biomarkers of Inflammation and Liver Function

IL-32 did not correlate with procalcitonin, CRP, IL-6, or immune cell count (Table 3). Positive correlations were observed with bilirubin, alanine aminotransferase (ALT), aspartate aminotransferase (AST), and gamma-glutamyl transferase (GGT) but not with albumin (Table 3).

The association of IL-32 with liver function may be a confounding factor. The upper limit of normal for AST and ALT is <50 U/L for men and <35 U/L for women. Using these cut-offs for ALT, 30% of men and 42% of women had abnormal values. Using these cut-offs for AST, 40% of men and 53% of women had abnormal values. Total bilirubin was above 0.2 mg/dL (normal value < 0.2 mg/dL) in all but one of the male and female patients. The normal value for GGT is <40 U/L in women and <60 U/L in men, and 80% of men and 84% of women had elevated levels. Albumin < 35 g/L, defined as low, was found in 3% of male and 8% of female patients. This shows that almost all of our patients had normal hepatic synthesis function, whereas the levels of AST, ALT, GGT, and bilirubin were increased in a significant number of patients.

### 3.6. Plasma IL-32 Levels and Survival

**Figure 4:**
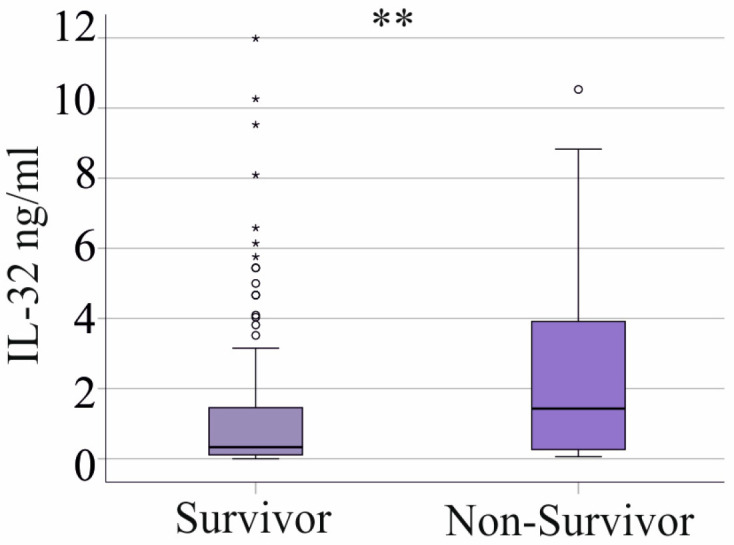
Association of plasma IL-32 levels with survival. Outliers are represented by circles and small asterisks. Mann–Whitney U test: ** *p* < 0.01.

Plasma IL-32 levels were elevated in the 28 non-surviving patients (27 patients with septic shock and 1 patient with sepsis excluding those with cirrhosis (Table S1)) compared to the survivors (Figure 4). The area under the receiver operating characteristic (AUROC) curve was 0.681 (*p* = 0.003), indicating that IL-32 is an acceptable but not an excellent marker for distinguishing between surviving and non-surviving patients with sepsis/septic shock.

Notably, 27 patients with septic shock and 1 patient with sepsis did not survive (Table S1). In the SIRS cohort, all patients survived.

In the group of SIRS/sepsis patients with liver cirrhosis, 14 patients did not survive, but the serum IL-32 levels of survivors and non-survivors were similar (*p* > 0.05).

## 4. Discussion

This study demonstrated that plasma IL-32 levels are lower in patients with SIRS/sepsis compared to the healthy controls. Notably, higher IL-32 levels in SIRS/sepsis patients were associated with increased mortality. These findings suggest that the reduction in IL-32 in severe disease may play a protective role.

IL-32 is known to be upregulated in the serum of patients with liver disease [33]. Elevated circulating IL-32 levels have been reported in individuals with non-alcoholic fatty liver disease and hepatitis B virus infection compared to healthy controls [33,34]. In non-alcoholic steatohepatitis and chronic hepatitis B, hepatic IL-32 expression is also increased [13,14]. In patients with hepatitis C infection, hepatic IL-32 mRNA expression has been positively correlated with inflammation, fibrosis scores, and serum alanine aminotransferase levels [12]. Experimental studies further suggest that IL-32 plays a role in liver pathology. The downregulation of IL-32 in hepatocytes has been shown to reduce collagen synthesis and triglyceride accumulation, indicating that elevated IL-32 contributes to liver steatosis and fibrosis [35]. In our study, plasma IL-32 levels were significantly higher in SIRS/sepsis patients with liver cirrhosis. To our knowledge, this is the first study demonstrating that IL-32 remains elevated in septic patients with underlying liver cirrhosis. These findings suggest a potential role for IL-32 in the interplay between systemic inflammation and liver dysfunction in critically ill patients, warranting further investigation.

The positive correlation between IL-32 and markers of liver injury—such as bilirubin, gamma-glutamyl transferase, and aminotransferases—in patients without liver cirrhosis suggests that the association between IL-32 expression and liver damage extends to critical illness. Assessing liver function in patients with sepsis is a challenge, and higher levels of liver enzymes may be induced by drugs, muscle breakdown, and inflammation. Albumin is a marker of liver synthetic function and may be better for the diagnosis of liver disease in patients with sepsis [36,37]. IL-32 did not correlate with albumin levels and the correlation with bilirubin, AST, ALT, and GGT does not necessarily indicate an association of IL-32 with liver disease.

The cellular source of elevated plasma IL-32 in liver cirrhosis remains unclear. Liver cirrhosis is associated with increased mortality risk [38], but the potential role of IL-32 in this context is yet to be determined. In our study, plasma IL-32 levels in patients with liver cirrhosis were not associated with mortality. While these findings suggest that IL-32 may not directly influence survival in cirrhotic patients with sepsis, the small sample size limits definitive conclusions. Further research is needed to clarify the underlying mechanisms and the potential impact of IL-32 in liver disease and critical illness.

Plasma IL-32 levels were lower in patients with SIRS/sepsis compared to healthy controls. This reduction became statistically significant only after excluding patients with liver cirrhosis, with increased systemic IL-32 concentrations. A similar decrease in serum IL-32 levels has been reported in SARS-CoV-2 infection [18]. In line with this observation [18], our study found that patients with COVID-19 had lower plasma IL-32 levels than the healthy controls. When comparing plasma IL-32 levels between SIRS/sepsis patients with and without SARS-CoV-2 infection, no significant differences were observed. This finding suggests that the reduction in IL-32 levels in COVID-19 patients [18] is likely a consequence of critical illness rather than a direct effect of viral infection. Further research is needed to elucidate the underlying mechanisms regulating IL-32 expression in sepsis and COVID-19.

The decline in plasma IL-32 levels observed in SARS-CoV-2 infection contrasts with findings in other viral infections. In response to influenza virus infection, serum IL-32 levels were significantly elevated [39], and the infection of lung epithelial cells resulted in a substantial increase in IL-32 release into the supernatant [39]. Similarly, HIV infection has been shown to upregulate IL-32 protein expression, with HIV-infected individuals exhibiting higher serum IL-32 levels compared to healthy controls [40]. Additionally, IL-32 has been reported to interfere with HIV replication, suggesting a potential antiviral role [40].

The reason for reduced plasma IL-32 levels in SARS-CoV-2 infection remains unclear. Given that a similar reduction was observed in patients with sepsis, it is plausible that disease severity, rather than the viral infection itself, contributes to the decrease in IL-32 levels. Further studies are needed to elucidate the mechanisms regulating IL-32 expression in severe infections and to determine whether this reduction has clinical significance.

In patients with SIRS/sepsis, plasma IL-32 levels were not associated with disease severity. Similarly, COVID-19 patients with mild, moderate, and severe disease exhibited a comparable reduction in IL-32 levels relative to healthy controls [18]. Within the SIRS/sepsis cohort, IL-32 plasma concentrations remained similar across patients diagnosed with SIRS, sepsis, and septic shock, whose disease severity increases in this order [30]. Furthermore, the need for mechanical ventilation, vasopressor therapy, or dialysis in mainly septic shock patients did not significantly impact IL-32 levels.

These findings suggest that the decline in IL-32 observed in patients with inflammatory diseases occurs independently of disease severity. Further research is needed to determine the mechanisms regulating IL-32 expression and whether this reduction plays any role in critically ill patients.

IL-32 is a well-characterized pro-inflammatory cytokine that stimulates the expression of inflammatory mediators in immune cells [17]. However, in our SIRS/sepsis cohort, plasma IL-32 levels did not correlate with inflammatory markers or immune cell counts. Given that T cells are a primary source of IL-32 [41], the lymphopenia observed in sepsis [42] may contribute to the reduced systemic IL-32 levels. While lymphopenia is associated with increased mortality [42], higher IL-32 levels were observed in non-surviving SIRS/sepsis patients. These findings highlight the need for further research to elucidate the mechanisms regulating IL-32 plasma levels in sepsis. Non-surviving patients had higher plasma IL-32 levels than survivors. A similar trend was observed in COVID-19 survivors [19], whereas another study found no association between IL-32 levels and mortality in hospitalized COVID-19 patients [20].

Sex, age, and body mass index are potential confounding factors in observational studies. In our cohort, plasma IL-32 levels did not differ between male and female patients. However, IL-32 levels showed a negative correlation with age, indicating that older patients had lower plasma IL-32 concentrations. The R-squared value for this correlation was 0.048, suggesting that age accounts for only a small proportion of the variance in IL-32 levels. Obesity has been linked to increased IL-32 expression in visceral and subcutaneous adipose tissue as well as in peripheral blood mononuclear cells [43]. Additionally, serum IL-32 levels have been reported to be elevated in obese individuals [44]. Despite these associations, we found no correlation between plasma IL-32 levels and body mass index in SIRS/sepsis patients.

This study has certain limitations. The ELISA used to measure plasma IL-32 levels does not differentiate between IL-32 isoforms. IL-32 exists in nine alternatively spliced isoforms, all derived from the pre-mRNA of the IL-32γ isoform. Among these, IL-32γ, IL-32α, and IL-32β are the longest, with IL-32γ being the most biologically active [45]. However, other studies have reported that recombinant IL-32θ exhibits the highest activity in both immune and non-immune cells [46].

As a result, it remains unclear which specific IL-32 isoforms were measured in our patient cohort and to what extent they are biologically active. Further studies are needed to distinguish the functional roles of individual IL-32 isoforms in critically ill patients. Plasma was only collected on admission to the intensive care unit, and we cannot provide any information on IL-32 plasma levels during the disease. In addition, the plasma was stored for almost 6 years, and there are currently no studies that have determined the stability of IL-32 during storage.

## 5. Conclusions

This study identified a reduction in plasma IL-32 levels in patients with SIRS/sepsis compared to the healthy controls. Of clinical relevance, non-survivors exhibited elevated IL-32 levels, suggesting that its decline may serve as a protective mechanism in critical illness. In septic patients with liver cirrhosis, plasma IL-32 concentrations were increased. The potential role of IL-32 in the increased mortality risk of sepsis patients with chronic liver disease remains unclear. Further research is needed to determine whether IL-32 actively contributes to disease progression or reflects an adaptive response to severe inflammation.

## Figures and Tables

**Table 1:** Characteristics of controls and SIRS/sepsis patients excluding 34 patients with liver cirrhosis. Numbers in superscript refer to patients for whom these data were available when data were not collected from the entire cohort. Statistical tests used: Mann–Whitney U test and chi-squared test. ** *p* < 0.01, *** *p* < 0.001.

Parameters	SIRS/Sepsis	Controls
Males/Females	110/42	19/21 **
Age, years	60 (21–93)	54 (21–63) ***
Body mass index, kg/m^2^	26.7 (15.4–55.6) ^148^	not defined
SIRS/Sepsis/Septic shock	39/37/76	not defined
C-reactive protein, mg/L	178 (4–697)	not defined
Procalcitonin, ng/mL	1.48 (0.05–270.00) ^148^	not defined
Leukocytes, n/nL	10.40 (0.06–246.94)	not defined
Neutrophils, n/nL	7.94 (0–70.20) ^146^	not defined
Basophils, n/nL	0.04 (0–0.90) ^147^	not defined
Eosinophils, n/nL	0.09 (0–8.80) ^147^	not defined
Monocytes, n/nL	0.76 (0–45.00) ^147^	not defined
Lymphocytes, n/nL	0.96 (0.08–28.60) ^147^	not defined
Immature granulocytes, n/nL	0.15 (0–7.25) ^147^	not defined
Total bilirubin, mg/dL	0.80 (0.10–23.90) ^144^	not defined
Albumin, g/L	24.1 (6.0–42.0) ^141^	not defined
Aspartate aminotransferase, U/L	43 (6–3252) ^139^	not defined
Alanine aminotransferase, U/L	32 (5–889) ^139^	not defined
Gamma-glutamyl transferase, U/L	137 (11–1266) ^125^	not defined

**Table 2:** Comparison of plasma IL-32 levels between patients on dialysis, mechanical ventilation, and vasopressor therapy and patients without these conditions. Patients with liver cirrhosis were excluded. The number of patients and corresponding *p*-values are presented. Statistical test used: Mann–Whitney U test.

Intervention/Drug		
	Number	*p*-Value
Dialysis	51	0.532
Ventilation	91	0.348
Vasopressor therapy	96	0.733

**Table 3:** Correlation coefficients (r) for plasma IL-32 levels and its associations with clinical markers of inflammation and liver disease. Statistical test used: Spearman correlation. * *p* < 0.05, ** *p* < 0.01, *** *p* < 0.001.

Biomarker of Inflammation	SIRS/Sepsis Without Liver Cirrhosis
Procalcitonin	0.095
C-reactive protein	−0.045
IL-6	0.140
Leukocytes	0.098
Neutrophils	0.075
Basophils	0.038
Eosinophils	−0.011
Monocytes	0.011
Lymphocytes	−0.049
Immature granulocytes	0.138
**Biomarker of Liver Disease**	**SIRS/Sepsis Without Liver Cirrhosis**
Total bilirubin, mg/dL	0.324 ***
Albumin, g/L	0.025
Aspartate aminotransferase, U/L	0.265 **
Alanine aminotransferase, U/L	0.189 *
Gamma-glutamyl transferase, U/L	0.245 **

## Data Availability

Data supporting reported results can be obtained from the corresponding author.

## Supplementary Materials

The following supporting information can be downloaded at: https://www.mdpi.com/article/10.3390/biomedicines13030750/s1, Figure S1: Representative standard curve of the IL-32 ELISA. Table S1: Characteristics of SIRS, sepsis, and septic shock patients, excluding 34 patients with liver cirrhosis.

## Abbreviations

The following abbreviations are used in this manuscript:

ALT	Alanine aminotransferase
AST	Aspartate aminotransferase
ARDS	Acute respiratory distress syndrome
CRP	C-reactive protein
GGT	Gamma-glutamyl transferase
HIV	Human immunodeficiency virus
IL	Interleukin
NK	Natural killer
SARS-CoV-2	Severe acute respiratory syndrome coronavirus 2
