# Supplementary material for: Lower Plasma IL-32 Levels Linked to Better Survival in Sepsis
Source: Biomedicines. 2025 Mar 19;13(3):750. doi: 10.3390/biomedicines13030750 (PMC11940173; doi:10.3390/biomedicines13030750)
Supplement: Supplementary file 1 [file biomedicines-13-00750-s001.zip › biomedicines-3502093-supplementary.pdf]

Figure S1. Representative standard curve of the IL-32 ELISA.

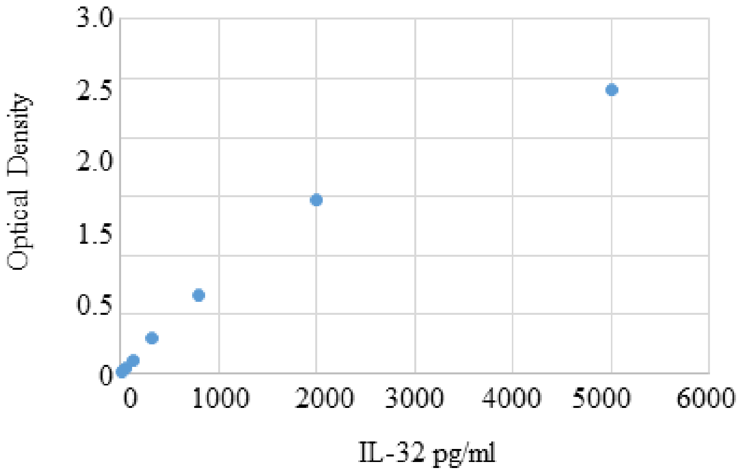

Table S1. Characteristics of SIRS, sepsis and septic shock patients excluding 34 patients with liver cirrhosis. Numbers in superscript refer to patients for whom these data were available when data were not collected from the entire cohort. Statistical tests used: Mann-Whitney U test and Chi-square test. * $p < 0.05$, ** and $^{\&\&}$ $p < 0.01$, *** and $^{\%\%\%}$ $p < 0.001$, and significant different values were labeled with identical symbols. The Chi-square test does not include a post-hoc test, and the p-values refer to the comparison of the three groups.

Parameters	SIRS	Sepsis	Septic Shock
Males/Females	29/10	24/13	57/19
Age (years)	57 (24 – 83)	53 (28 – 83)*	63 (21 – 93)*
Body Mass Index (kg/m^2)	25.4 (18.3 – 41.0)^{38}	26.8 (18.4 – 54.5)^{37}	28.5 (15.4 – 55.6)^{73}
C-reactive protein mg/l	177 (4 – 486)	207 (40 – 503)	174 (20 – 697)
Procalcitonin ng/ml	2.00 (0.05 – 270.00)^{39}	1.00 (0.06 – 112.27)^{36}	1.61 (0.08 – 114.40)^{73}
Leukocytes n/nl	9.73 (0.06 – 37.38)	11.40 (0.28 – 40.44)	10.19 (0.32 – 246.94)
Neutrophils n/nl	7.23 (1.93 – 29.73)^{37}	9.03 (0 – 70.20)^{37}	8.23 (0 – 48.40)^{72}
Basophils n/nl	0.04 (0 – 0.38)^{37}	0.05 (0 – 0.90)^{37}	0.03 (0 – 0.60)^{73}
Eosinophils n/nl	0.07 (0 – 0.96)^{37}	0.06 (0 – 1.75)^{37}	0.10 (0 – 8.80)^{73}
Monocytes n/nl	0.59 (0 – 3.59)^{37}	0.88 (0 – 45.00)^{37}	0.71 (0 – 10.90)^{73}
Lymphocytes n/nl	0.87 (0.10 –2.79)^{37}	1.20 (0.29 –16.80)^{37}	0.94 (0.08 –28.60)^{73}
Immature Granulocytes n/nl	0.06 (0 – 2.00)$^{37\ \&\&,\%\%\%}$	0.21 (0 – 6.19)$^{37\&\&}$	0.24 (0 – 7.25)$^{73\ \%\%\%}$
Total Bilirubin mg/dl	0.80 (0.10 – 18.80)^{37}	0.80 (0.2 – 18.60)^{37}	0.70 (0.20 – 23.90)^{60}
Albumin g/l	22.3 (13.0 – 33.1)^{35}	24.1 (15.5 – 33.9)^{37}	24.7 (6.3 – 42.0)^{69}
Aspartate Aminotransferase U/l	42 (6 – 3252)^{38}	40 (8– 603)^{35}	44 (8– 1703)^{66}
Alanine Aminotransferase U/l	32 (5 – 889)^{37}	31 (7 – 559)^{33}	32 (6 – 770)^{69}
Gamma-Glutamyl Transferase U/l	171 (30 – 1093)^{33}	142 (27 – 467)^{33}	115 (11 – 1266)^{59}
**Interventions**			
Dialysis	5	5	41 ***
Ventilation	7	16	68 ***
Vasopressor therapy	10	16	70 ***
**Non-survival**	0	1	27 ***
**Infections**			
SARS-CoV-2	0	7	21**
Gram negative	9	2	8
Gram positive	6	3	12
Gram positive and negative	0	0	4
Urosepsis	6	4	6
Pulmonary Infection	4	6	33**
**Underlying Diseases**			
Pancreatitis	12	14	15
Cholangiosepsis	3	2	4
